# Association of the *ST3GAL4* rs11220462 polymorphism and serum lipid levels in the Mulao and Han populations

**DOI:** 10.1186/1476-511X-13-123

**Published:** 2014-08-03

**Authors:** Quan-Zhen Lin, Rui-Xing Yin, Tao Guo, Jian Wu, Jia-Qi Sun, Shao-Wen Shen, Guang-Yuan Shi, Jin-Zhen Wu, Cheng-Wu Liu, Shang-Ling Pan

**Affiliations:** 1 Department of Cardiology, Institute of Cardiovascular Diseases, the First Affiliated Hospital, Guangxi Medical University, 22 Shuangyong Road, Nanning 530021 Guangxi, People’s Republic of China; 2 Department of Pathophysiology, School of Premedical Sciences, Guangxi Medical University, Nanning 530021 Guangxi, People’s Republic of China

**Keywords:** Lipids, ST3 beta-galactoside alpha-2,3-sialytransferase 4, Single nucleotide polymorphism, Environmental factors

## Abstract

**Background:**

A previous genome-wide association study has displayed the association of the ST3 beta-galactoside alpha-2,3-sialytransferase 4 (ST3GAL4) gene variant and lipid traits in the individuals of European ancestry, but the reproducibility of this association has not been detected in the Chinese population. The present study was undertaken to detect the association of *ST3GAL4* rs11220462 single nucleotide polymorphism (SNP) and several environmental factors with serum lipid profiles in the Mulao and Han populations.

**Methods:**

A total of 700 unrelated individuals of Mulao nationality and 694 subjects of Han nationality were randomly selected from our previous stratified randomized samples. Genotypes of the SNP were determined via polymerase chain reaction and restriction fragment length polymorphism in combination with gel electrophoresis, and then verified by direct sequencing.

**Results:**

Serum apolipoprotein (Apo) B levels were higher and the ApoAI/ApoB ratio was lower in Mulao than in Han (*P* < 0.05-0.01). There were no significant differences in the genotypic and allelic frequencies of the *ST3GAL4* rs11220462 SNP between the two ethnic groups or between males and females. The A allele carriers in both Mulao males and females had higher total cholesterol (TC), low-density lipoprotein cholesterol (LDL-C), and ApoB levels than the A allele non-carriers (*P* < 0.05-0.01). The subjects with AA genotype in Han males but not in females had higher TC and triglyceride (TG) levels than the subjects with AG or GG genotype (*P* < 0.01 for each). Multiple linear regression analyses showed that the levels of TC, LDL-C and ApoB in Mulao females; TC and LDL-C in Mulao males; and TC in Han males were correlated with the genotypes (*P <* 0.05-0.001). Serum lipid parameters were also associated with several environmental factors in both ethnic groups (*P* < 0.05 -0.001).

**Conclusions:**

The association of *ST3GAL4* rs11220462 SNP and serum lipid levels was different between the Mulao and Han populations, suggesting that there may be a racial/ethnic-specific association, and/or sex-specific association between the *ST3GAL4* rs11220462 SNP and serum lipid parameters in some ethnic groups.

## Background

It is well known that coronary artery disease (CAD) is the leading cause of morbidity and mortality globally [1,2]. Dyslipidemia, associated with elevating total cholesterol (TC) [3], triglyceride (TG) [4], low-density lipoprotein cholesterol (LDL-C) [5], and apolipoprotein (Apo) B [6], or low levels of high-density lipoprotein cholesterol (HDL-C) [7] and ApoAI [6], plays a significant role for CAD and is the main target for therapeutic intervention. The heritability estimates of the interindividual variations in serum lipid levels from both twin and family studies are in the range of 0.48-0.87 [8-11], suggesting a considerable genetic contribution. Therefore, the understanding of the association of genetic variants and serum lipid levels may provide new insights into target preventive intervention for CAD.

Over the years, candidate gene studies as well as genome-wide association studies (GWASs) have enabled the identification of thousands of single nucleotide polymorphisms (SNPs) associated with complex human diseases and traits. These studies based on large numbers of normolipidemic individuals indicated that considerable new SNPs had replicable modest associations with plasma concentrations of TC, TG, LDL-C, and HDL-C [12-18]. The ST3 beta-galactoside alpha-2,3-sialytransferase 4 (ST3GAL4, as known as SIAT4) rs11220462 (http://www.ncbi.nlm.nih.gov/gene/6484) is one of the identified SNPs in recent years. *ST3GAL4* locates on chromosome 11q24.2 [15] and encodes a member of the glycosyltransferase 29 family, a group of enzymes involved in protein glycosylation. The products of *ST3GAL4* were associated with increasing risk of cirrhosis, type 2 diabetes and cardiovascular disease by influencing liver enzyme concentrations [19]. Interestingly, *ST3GAL4* has been correlated to endothelial dysfunction and the synthesis of E-selectin ligands, which were associated with metabolic syndrome and coronary artery calcification [20,21]. In a previous GWAS, the SNP of *ST3GAL4* rs11220462 has been associated with TC and LDL-C concentrations in European population [15]. But the reproducibility and repeatability of this association have not been carried out in the Chinese population so far. Therefore, it would be attractive to characterize the association between the rs11220462 SNP and serum lipid levels in the Chinese population.

China is a multiethnic country. Han nationality is the largest one of 56 ethnic groups, and Mulao nationality is the twenty-ninth largest (with population of 207,352 in 2000) among the 55 minorities. Ninety percent of them live in the Luocheng Mulao Autonomous Country, Guangxi Zhuang Autonomous Region, People’s Republic of China. The history of this minority can be traced back to the Jin Dynasty (AD 265–420). It is well known that the people of Mulao are the descendants of the ancient Baiyue tribe in south China. In a previous study, Xu et al. [22] showed that the genetic relationship between Mulao nationality and other minorities in Guangxi was much closer than that between Mulao and Han or Uighur nationality. To the best of our knowledge, the association between *ST3GAL4* rs11220462 SNP and serum lipid levels has not been previously explored in the Chinese population. Therefore, the aim of the present study was to detect the association of *ST3GAL4* rs11220462 SNP and several environmental factors with serum lipid traits in the Guangxi Mulao and Han populations.

## Results

### General characteristics and serum lipid levels

The baseline characteristics and serum lipid levels of the Mulao and Han populations are presented in Table 1. The levels of weight, body mass index (BMI), the ratio of ApoAI to ApoB and the percentages of individuals who smoked cigarettes were lower in Mulao than in Han (*P* < 0.05-0.001), whereas the levels of ApoB were higher in Mulao than in Han (*P* < 0.05). There were no significant differences in the levels of age structure, body height, waist circumference, systolic blood pressure, diastolic blood pressure, pulse pressure, blood glucose, TC, TG, HDL-C, LDL-C, ApoAI; the percentages of subjects who consumed alcohol; and the ratio of male to female between the two ethnic groups (*P* > 0.05 for all).

**Table 1:** Comparison of demography, lifestyle and serum lipid levels between the Mulao and Han populations

**Parameter**	**Han**	**Mulao**	***t *** **(*χ***^***2***^**)**	***P***
Number	694	700		
Male/female	331/363	337/363	0.280	0.867
Age (years)	52.26 ± 15.02	52.56 ± 15.04	0.379	0.705
Height (cm)	154.93 ± 8.12	155.49 ± 8.07	1.291	0.197
Weight (kg)	54.02 ± 9.12	53.02 ± 9.40	−1.998	0.046
Body mass index (kg/m^2^)	22.48 ± 3.39	21.87 ± 3.08	−3.553	0.000
Waist circumference (cm)	75.62 ± 7.91	75.13 ± 8.67	−1.099	0.272
Cigarette smoking [n (%)]				
Nonsmoker	480 (69.2)	512 (73.1)		
≤ 20 cigarettes/day	192 (27.6)	151 (21.6)	9.728	0.008
> 20 cigarettes/day	22 (3.2)	37 (5.3)		
Alcohol consumption [n (%)]				
Nondrinker	516 (74.3)	515 (73.6)		
≤ 25g/day	81 (11.7)	70 (10.0)	2.305	0.316
> 25g/day	97 (14.0)	115 (16.4)		
Systolic blood pressure (mmHg)	130.51 ± 19.29	129.25 ± 21.52	−1.150	0.250
Diastolic blood pressure (mmHg)	82.14 ± 11.01	80.97 ± 11.89	1.912	0.056
Pulse pressure (mmHg)	48.37 ± 15.02	48.28 ± 15.82	−0.102	0.918
Blood glucose (mmol/L)	5.97 ± 1.58	5.97 ± 1.52	−0.034	0.973
Total cholesterol (mmol/L)	5.03 ± 0.94	4.98 ± 1.08	−0.931	0.352
Triglyceride (mmol/L)	1.08 (0.86)	1.07 (0.79)	−0.882	0.378
HDL-C (mmol/L)	1.75 ± 0.54	1.75 ± 0 .45	0.096	0.924
LDL-C (mmol/L)	2.90 ± 0.80	2.93 ± 0.83	0.088	0.930
Apolipoprotein (Apo) AI (g/L)	1.35 ± 0.60	1.32 ± 0.40	−1.815	0.070
ApoB (g/L)	0.85 ± 0.18	0.96 ± 0.54	5.196	0.000
ApoAI/ApoB	1.65 ± 0 .48	1.57 ± 0.74	−2.298	0.022

*TC*, total cholesterol; *TG*, triglyceride; *HDL-C*, high-density lipoprotein cholesterol; *LDL-C*, low-density lipoprotein cholesterol; *ApoAI*, apolipoprotein AI; *ApoB*, apolipoprotein B. The quantitative variables were presented as mean ± standard deviation and their difference between the groups was determined by the *t*-test. The values of triglyceride were presented as median (interquartile range), and their difference between the groups was determined by the Wilcoxon-Mann–Whitney test. The difference in Percentages of cigarette smoking and alcohol consumption between the groups was determined by Chi-square -test.

### Genotypic and allelic frequencies

The genotypic and allelic frequencies of the rs11220462 SNP in the both ethnic groups are shown in Table 2. The frequencies of G and A alleles were 64.5% and 35.5% in Mulao; and 61.7% and 38.3% in Han (*P* > 0.05); respectively. The frequencies of GG, AG and AA genotypes were 40.7%, 48.1% and 11.2% in Mulao, and 36.4%, 50.6% and 13.0% in Han (*P* > 0.05); respectively. There was no significant difference in the genotypic and allelic frequencies between males and females in the both ethnic groups (*P* > 0.05 for all).

**Table 2:** Comparison of the genotypic and allelic frequencies between the Mulao and Han populations [n (%)]

**Group**	**n**	**Genotype**			**Allele**		**HWE**
		**GG**	**AG**	**AA**	**G**	**A**	**(*P *** **value)**
Han	694	253 (36.4)	351 (50.6)	90 (13.0)	857 (61.7)	531 (38.3)	0.063
Mulao	700	281 (40.1)	341 (48.7)	78 (11.2)	903 (64.5)	497 (35.5)	0.092
*χ*^*2*^	-	2.444			2.275		
*P*	-	0.295			0.131		
Han							
Male	331	119 (36.0)	169 (51.0)	43 (13.0)	407 (61.5)	255 (38.5)	0.156
Female	363	134 (36.9)	182 (50.1)	47 (13.0)	450 (62.0)	276 (38.0)	0.224
*χ*^*2*^	-	0.073			0.037		
*P*	-	0.964			0.847		
Mulao							
Male	337	127 (37.6)	165 (49.0)	45 (13.4)	419 (62.2)	255 (37.8)	0.453
Female	363	154 (42.4)	176 (48.5)	33 (9.1)	484 (66.7)	242 (33.3)	0.083
*χ*^*2*^	-	3.835			3.092		
*P*	-	0.147			0.079		

*HWE*, Hardy–Weinberg equilibrium. Difference in genotype or allele distribution between the groups was tested by the Chi-square test; a standard goodness-of-fit test was used to test the Hardy–Weinberg equilibrium.

### Genotypes and serum lipid levels

As shown in Table 3, the levels of TC and LDL-C in Mulao but not in Han were different among the AA, AG and GG genotypes after adjusting age, sex, height, BMI, blood pressure, cigarette smoking, alcohol consumption and blood glucose (*P* < 0.01 for each); the A allele carriers had higher levels of TC and LDL-C than the A allele non-carriers. In the subgroup analyses, the levels of TC and TG in Han males but not in females were different among the genotypes (*P* < 0.01 for each), the subjects with AA genotype had higher TC and TG levels than the subjects with AG or GG genotype. For the Mulao population, the levels of TC, LDL-C and ApoB in both males and females were also different among the genotypes (*P* < 0.05-0.01), the A allele carriers had higher serum TC, LDL-C and ApoB levels than the A allele non-carriers.

**Table 3:** Comparison of serum lipid levels among the genotypes in the Mulao and Han populations

**Genotype**	**n**	**TC**	**TG**	**HDL-C**	**LDL-C**	**ApoAI**	**ApoB**	**ApoAI/ApoB**
		**(mmol/L)**	**(mmol/L)**	**(mmol/L)**	**(mmol/L)**	**(g/L)**	**(g/L)**	
Han								
GG	253	5.00 ± 0.96	1.08 (1.07)	1.71 ± 0.55	2.85 ± 0.78	1.36 ± 0.29	0.87 ± 0.19	1.64 ± 0.53
AG	351	5.05 ± 0.92	1.05 (0.74)	1.77 ± 0.60	2.95 ± 0.81	1.35 ± 0.23	0.85 ± 0.18	1.65 ± 0.44
AA	90	5.10 ± 0.99	1.08 (1.09)	1.73 ± 0.43	2.96 ± 0.81	1.36 ± 0.30	0.85 ± 0.19	1.68 ± 0.50
*F*	-	0.519	4.963	1.041	0.309	0.121	0.391	0.437
*P*	-	0.595	0.084	0.353	0.734	0.886	0.676	0.646
Mulao								
GG	286	4.80 ± 1.18	1.04 (0.84)	1.73 ± 0.41	2.82 ± 0.82	1.31 ± 0.41	0.89 ± 0.25	1.57 ± 0.70
AG	344	5.09 ± 1.01	1.10 (0.73)	1.77 ± 0.49	2.98 ± 0.83	1.33 ± 0.39	0.90 ± 0.53	1.58 ± 0.79
AA	76	5.17 ± 0.89	1.15 (0.77)	1.74 ± 0.41	3.09 ± 0.80	1.30 ± 0.42	0.98 ± 0.61	1.53 ± 0.64
*F*	-	7.378	1.784	0.910	5.405	0.056	0.587	0.086
*P*	-	0.001	0.410	0.403	0.005	0.946	0.556	0.918
Han/male								
GG	120	5.00 ± 0.97	1.32 (1.18)	1.65 ± 0.42	2.90 ± 0.73	1.36 ± 0.32	0.91 ± 0.19	1.57 ± 0.58
AG	182	5.20 ± 0.73	1.09 (0.72)	1.73 ± 0.35	3.02 ± 0.68	1.37 ± 0.23	0.90 ± 0.15	1.57 ± 0.35
AA	29	5.41 ± 0.95	1.36 (0.47)	1.70 ± 0.69	3.04 ± 0.86	1.41 ± 0.39	0.89 ± 0.17	1.66 ± 0.58
*F*	-	7.255	12.933	1.734	1.422	2.147	0.736	1.304
*P*	-	0.001	0.002	0.178	0.243	0.118	0.480	0.273
Han/female								
GG	134	4.99 ± 0.96	0.96 (0.38)	1.80 ± 0.43	2.88 ± 0.83	1.35 ± 0.25	0.83 ± 0.17	1.70 ± 0.48
AG	182	4.90 ± 1.05	0.98 (0.79)	1.81 ± 0.76	2.88 ± 0.91	1.32 ± 0.23	0.81 ± 0.20	1.73 ± 0.51
AA	47	4.80 ± 0.94	1.07 (0.59)	1.71 ± 0.38	2.88 ± 0.77	1.32 ± 0.18	0.81 ± 0.20	1.70 ± 0.40
*F*	-	1.215	3.999	0.648	0.113	0.305	1.166	0.973
*P*	-	0.298	0.135	0.524	0.894	0.737	0.313	0.379
Mulao/male								
GG	130	4.77 ± 1.17	1.04 (1.05)	1.75 ± 0.44	2.72 ± 0.82	1.31 ± 0.46	0.88 ± 0.17	1.59 ± 0.68
AG	167	5.04 ± 0.85	1.17 (1.05)	1.75 ± 0.57	2.96 ± 0.73	1.36 ± 0.39	0.90 ± 0.66	1.52 ± 0.69
AA	43	5.08 ± 0.84	1.15 (0.77)	1.66 ± 0.37	2.97 ± 0.81	1.23 ± 0.42	0.91 ± 0.47	1.42 ± 0.51
*F*	-	5.108	2.476	0.839	3.230	1.478	3.182	1.166
*P*	-	0.007	0.290	0.433	0.041	0.230	0.043	0.313
Mulao/female								
GG	156	4.82 ± 1.18	1.04 (0.64)	1.72 ± 0.39	2.90 ± 0.82	1.32 ± 0.37	0.88 ± 0.35	1.56 ± 0.71
AG	177	5.03 ± 1.14	1.01 (0.66)	1.78 ± 0.41	3.00 ± 0.91	1.30 ± 0.38	0.93 ± 0.33	1.64 ± 0.87
AA	33	5.40 ± 0.92	1.04 (0.73)	1.84 ± 0.44	3.29 ± 0.89	1.39 ± 0.41	1.04 ± 0.70	1.68 ± 0.76
*F*	-	4.376	0.393	1.251	3.097	0.891	3.678	0.289
*P*	-	0.023	0.822	0.287	0.049	0.411	0.026	0.749

*TC*, total cholesterol; *TG*, triglycerides; *HDL-C*, high-density lipoprotein cholesterol; *LDL-C*, low-density lipoprotein cholesterol; *ApoAI*, Apo lipoprotein AI; *ApoB*, Apo lipoprotein B. The association of genotypes and serum lipid parameters was tested by analysis of covariance (co-variables include sex, age, body mass index, blood pressure, alcohol consumption, cigarette smoking and blood glucose). The value of TG was presented as median (interquartile range), and their difference among the genotypes was determined the Wilcoxon-Mann–Whitney test.

### Risk factors for serum lipid parameters

The risk factors for serum lipid parameters in Mulao and Han are shown in Table 4. The levels of TC and LDL-C in Mulao but not in Han were correlated with the genotypes (*P* < 0.05 for each). Serum TC and LDL-C levels in both Mulao males and females, ApoB levels in Mulao females, and TC levels in Han males were correlated with genotypes (*P* < 0.05 for all, Table 5); respectively. Serum lipid parameters were also correlated with several environmental factors such as age, gender, height, weight, BMI, waist circumference, alcohol consumption, cigarette smoking, blood pressure and blood glucose in both ethnic groups (*P* < 0.05-0.001; Tables 4 and 5).

**Table 4:** Relative risk factors for serum lipid parameters in the Mulao and Han populations

**Lipid parameter**	**Risk factor**	**Unstandardized coefficient**	**Standard error**	**Standardized coefficient**	***t***	***P***
Han/Mulao						
TC	Age	0.010	0.002	0.168	5.242	0.000
	Genotype	−0.133	0.040	−0.087	−3.359	0.001
	Alcohol consumption	0.080	0.036	0.058	2.232	0.026
	Systolic blood pressure	0.004	0.001	0.083	2.867	0.004
	Body mass index	0.046	0.008	0.147	5.530	0.000
TG	Waist circumference	0.029	0.006	0.196	4.695	0.000
	Alcohol consumption	0.185	0.044	0.113	4.171	0.000
	Blood glucose	0.068	0.020	0.086	3.375	0.001
	Diastolic blood pressure	0.010	0.003	0.097	3.681	0.000
	Weight	0.095	0.025	0.723	3.796	0.000
	Height	−0.066	0.018	−0.442	−3.646	0.000
HDL-C	Waist circumference	−0.007	0.002	−0.109	−2.695	0.007
	Alcohol consumption	0.104	0.019	0.154	5.586	0.000
	Weight	−0.009	0.002	−0.175	−4.127	0.000
LDL-C	Body mass index	0.042	0.007	0.169	6.330	0.000
	Age	0.008	0.002	0.152	5.350	0.000
	Genotype	−0.083	0.032	−0.068	−2.611	0.009
	Alcohol consumption	−0.081	0.029	−0.074	−2.804	0.005
	Systolic blood pressure	0.002	0.001	0.059	2.044	0.041
ApoAI	Ethnic group	0.040	0.017	0.059	2.265	0.024
	Gender	0.059	0.021	0.087	2.774	0.006
	Age	0.001	0.001	0.052	2.007	0.045
	Alcohol consumption	0.130	0.014	0.284	9.210	0.000
	Waist circumference	−0.005	0.001	−0.125	−4.675	0.000
ApoB	Ethnic group	−0.122	0.021	−0.149	−5.772	0.000
	Body mass index	0.010	0.005	0.079	2.168	0.030
	Waist circumference	0.007	0.002	0.134	3.695	0.000
	Pulse pressure	0.003	0.001	0.117	4.464	0.000
	Blood glucose	0.019	0.007	0.07	2.657	0.008
ApoAI/ApoB	Ethnic group	0.103	0.032	0.083	3.256	0.001
	Gender	0.202	0.044	0.162	4.553	0.000
	Waist circumference	−0.013	0.003	−0.173	−4.086	0.000
	Blood glucose	−0.031	0.011	−0.077	−2.957	0.003
	Alcohol consumption	0.132	0.026	0.156	5.125	0.000
	Height	0.014	0.003	0.178	4.929	0.000
	Weight	−0.010	0.003	−0.145	−2.874	0.004
Han						
TC	Age	0.009	0.003	0.150	3.613	0.000
	Waist circumference	0.022	0.005	0.182	4.702	0.000
	Systolic blood pressure	0.005	0.002	0.110	2.642	0.008
	Alcohol consumption	0.136	0.051	0.104	2.653	0.008
	Height	−0.011	0.005	−0.098	−2.401	0.017
TG	Waist circumference	0.056	0.006	0.329	9.030	0.000
	Blood glucose	0.121	0.030	0.141	4.076	0.000
	Alcohol consumption	0.240	0.065	0.129	3.697	0.001
	Diastolic blood pressure	0.010	0.004	0.086	2.422	0.016
HDL-C	Alcohol consumption	0.118	0.033	0.158	3.583	0.000
	Waist circumference	−0.013	0.003	−0.195	−5.094	0.000
	Gender	0.222	0.055	0.206	4.067	0.000
	Cigarette smoking	0.142	0.047	0.141	3.022	0.003
	Age	0.003	0.001	0.080	2.178	0.030
LDL-C	Body mass index	0.044	0.009	0.185	5.085	0.000
	Age	0.012	0.002	0.233	6.394	0.000
ApoAI	Gender	0.065	0.026	0.125	2.515	0.012
	Alcohol consumption	0.142	0.015	0.394	9.480	0.000
	Weight	−0.007	0.001	−0.250	−6.288	0.000
	Cigarette smoking	0.092	0.021	0.189	4.308	0.000
ApoB	Waist circumference	0.006	0.001	0.254	4.621	0.000
	Systolic blood pressure	0.001	0.000	0.136	3.800	0.000
	Body mass index	0.007	0.003	0.120	2.207	0.028
	Blood glucose	0.014	0.004	0.118	3.411	0.001
	Gender	−0.060	0.016	−0.162	−3.712	0.000
	Height	−0.002	0.001	−0.105	−2.146	0.032
ApoAI/ApoB	Body mass index	−0.030	0.007	−0.214	−4.592	0.000
	Alcohol consumption	0.134	0.028	0.199	4.848	0.000
	Waist circumference	−0.011	0.003	−0.183	−3.850	0.000
	Gender	0.258	0.046	0.267	5.641	0.000
	Cigarette smoking	0.170	0.039	0.189	4.322	0.000
Mulao						
TC	Age	0.010	0.003	0.142	3.874	0.000
	Body mass index	0.053	0.013	0.150	4.087	0.000
	Genotype	−0.207	0.060	−0.126	−3.432	0.001
TG	Waist circumference	0.021	0.006	0.171	3.296	0.001
	Alcohol consumption	0.136	0.051	0.095	2.669	0.008
	Body mass index	0.055	0.018	0.158	3.059	0.002
	Diastolic blood pressure	0.010	0.003	0.107	2.888	0.004
HDL-C	Body mass index	−0.041	0.005	−0.277	−7.641	0.000
	Alcohol consumption	0.101	0.026	0.169	3.876	0.000
	Gender	0.084	0.039	0.093	2.126	0.034
LDL-C	Body mass index	0.046	0.010	0.170	4.610	0.000
	Age	0.007	0.002	0.127	3.467	0.001
	Genotype	−0.142	0.046	−0.113	−3.060	0.002
	Gender	0.149	0.061	0.090	2.440	0.015
ApoAI	Alcohol consumption	0.115	0.024	0.219	4.887	0.000
	Waist circumference	−0.005	0.002	−0.100	−2.632	0.009
	Gender	0.073	0.036	0.091	1.995	0.046
ApoB	Waist circumference	0.010	0.002	0.162	4.367	0.000
	Pulse pressure	0.005	0.001	0.140	3.782	0.000
ApoAI/ApoB	Waist circumference	−0.018	0.003	−0.217	−5.846	0.000
	Blood glucose	−0.049	0.018	−0.102	−2.761	0.006
	Alcohol consumption	0.083	0.036	0.085	2.301	0.022

*TC*, total cholesterol; *TG*, triglyceride; *HDL-C*, high-density lipoprotein cholesterol; *LDL-C*, low-density lipoprotein cholesterol; *ApoAI*, Apolipoprotein AI; *ApoB*, Apolipoprotein B. The correlative factors were determined by multivariable linear regression analysis with stepwise modeling.

**Table 5:** Relative risk factors for serum lipid parameters in males and females of the Mulao and Han populations

**Lipid parameter**	**Risk factor**	**Unstandardized coefficient**	**Standard error**	**Standardized coefficient**	***t***	***P***
Han/male						
TC	Diastolic blood pressure	0.019	0.004	0.241	4.461	0.000
	Genotype	−0.292	0.072	−0.219	−4.049	0.000
	Alcohol consumption	0.113	0.056	0.109	2.024	0.044
TG	Waist circumference	0.074	0.010	0.387	7.655	0.000
	Blood glucose	0.131	0.045	0.148	2.924	0.004
	Alcohol consumption	0.278	0.092	0.152	3.017	0.003
HDL-C	Waist circumference	−0.018	0.003	−0.335	−6.398	0.000
	Alcohol consumption	0.119	0.027	0.227	4.398	0.000
	Cigarette smoking	0.142	0.039	0.192	3.681	0.000
	Diastolic blood pressure	0.006	0.002	0.167	3.164	0.002
LDL-C	Body mass index	0.031	0.010	0.163	2.967	0.003
	Cigarette smoking	−0.187	0.068	−0.152	−2.763	0.006
	Age	0.005	0.003	0.113	2.063	0.040
ApoAI	Alcohol consumption	0.157	0.017	0.453	9.328	0.000
	Weight	−0.009	0.002	−0.290	−5.951	0.000
	Cigarette smoking	0.090	0.024	0.183	3.783	0.000
	Diastolic blood pressure	0.003	0.001	0.128	2.644	0.009
ApoB	Body mass index	0.013	0.003	0.292	4.625	0.000
	Waist circumference	0.004	0.001	0.190	2.986	0.003
	Blood glucose	0.013	0.005	0.136	2.691	0.008
ApoAI/ApoB	Alcohol consumption	0.143	0.028	0.253	5.140	0.000
	Weight	−0.055	0.013	−1.045	−4.357	0.000
	Cigarette smoking	0.130	0.039	0.162	3.360	0.001
	Height	0.040	0.012	0.645	3.442	0.001
	Systolic blood pressure	0.004	0.001	0.142	2.862	0.004
Han/female						
TC	Age	0.019	0.004	0.272	5.099	0.000
	Waist circumference	0.026	0.007	0.186	3.716	0.000
	Height	−0.025	0.009	−0.151	−2.862	0.004
TG	Waist circumference	0.042	0.008	0.268	5.104	0.000
	Blood glucose	0.098	0.040	0.123	2.427	0.016
	Diastolic blood pressure	0.013	0.005	0.121	2.312	0.021
HDL-C	Waist circumference	−0.010	0.005	−0.118	−2.208	0.028
LDL-C	Age	0.017	0.003	0.285	5.288	0.000
	Waist circumference	0.026	0.006	0.217	4.374	0.000
	Height	−0.018	0.007	−0.129	−2.447	0.015
	Alcohol consumption	−0.409	0.179	−0.113	−2.279	0.023
ApoAI	Body mass index	−0.011	0.004	−0.141	−2.628	0.009
	Cigarette smoking	0.187	0.068	0.145	2.760	0.006
	Diastolic blood pressure	−0.003	0.001	−0.131	−2.431	0.016
ApoB	Waist circumference	0.009	0.001	0.336	7.006	0.000
	Pulse pressure	0.003	0.001	0.194	3.746	0.000
	Height	−0.005	0.002	−0.150	−3.021	0.003
	Blood glucose	0.015	0.007	0.112	2.265	0.024
ApoAI/ApoB	Waist circumference	−0.018	0.003	−0.277	−5.576	0.000
	Systolic blood pressure	−0.006	0.001	−0.239	−4.698	0.000
	Cigarette smoking	0.399	0.125	0.154	3.194	0.002
	Height	0.009	0.004	0.118	2.371	0.018
Mulao/male						
TC	Body mass index	0.043	0.018	0.133	2.442	0.015
	Genotype	−0.161	0.081	−0.108	−1.976	0.049
TG	Waist circumference	0.025	0.011	0.171	2.280	0.023
	Diastolic blood pressure	0.015	0.006	0.139	2.588	0.010
	Body mass index	0.085	0.032	0.200	2.626	0.009
HDL-C	Body mass index	−0.050	0.008	−0.309	−5.993	0.000
	Alcohol consumption	0.110	0.029	0.195	3.775	0.000
LDL-C	Genotype	−0.153	0.063	−0.133	−2.435	0.015
ApoAI	Alcohol consumption	0.121	0.025	0.253	4.786	0.000
	Waist circumference	−0.006	0.002	−0.134	−2.526	0.012
ApoB	Pulse pressure	0.008	0.002	0.219	3.986	0.000
	Age	−0.004	0.002	−0.117	−2.145	0.033
	Body mass index	0.022	0.010	0.124	2.323	0.021
ApoAI/ApoB	Waist circumference	−0.017	0.004	−0.230	−4.361	0.000
	Alcohol consumption	0.136	0.040	0.180	3.411	0.001
Mulao/female						
TC	Age	0.014	0.004	0.185	3.652	0.000
	Genotype	−0.261	0.091	−0.145	−2.862	0.004
	Body mass index	0.060	0.019	0.160	3.168	0.002
TG	Waist circumference	0.024	0.005	0.232	4.529	0.000
HDL-C	Body mass index	−0.033	0.007	−0.248	−4.861	0.000
LDL-C	Age	0.012	0.003	0.213	4.285	0.000
	Body mass index	0.068	0.014	0.236	4.747	0.000
	Genotype	−0.261	0.091	−0.145	−2.862	0.004
ApoB	Age	0.004	0.002	0.127	2.470	0.014
	Waist circumference	0.013	0.003	0.189	3.740	0.000
	Blood glucose	0.056	0.020	0.143	2.769	0.006
	Genotype	0.098	0.042	0.117	2.339	0.020
ApoAI/ApoB	Waist circumference	−0.019	0.005	−0.191	−3.733	0.000
	Age	−0.008	0.003	−0.159	−3.114	0.002

*TC*, total cholesterol; *TG*, triglyceride; *HDL-C*, high-density lipoprotein cholesterol; *LDL-C*, low-density lipoprotein cholesterol; *ApoAI*, Apolipoprotein AI; *ApoB*, Apolipoprotein B. The correlative factors were determined by multivariable linear regression analysis with stepwise modeling.

## Discussion

In the current study, we showed that the levels of ApoB were higher whereas the ratio of ApoAI to ApoB was lower in Mulao than in Han (*P* < 0.05 for each). There were no significant differences in the levels of TC, TG, HDL-C, LDL-C and ApoAI between the two ethnic groups (*P* > 0.05 for all). In contrast with developed countries, China has experienced a considerable increase in the prevalence of CAD over the past several decades [23]. Evidence of the relationship between dyslipidemia and CAD has been established worldwide. It is well recognized that dyslipidemia is a multifactorial and complicated origin which combined by genetic factors with environmental factors including demographics, diet, alcohol consumption, cigarette smoking, obesity, exercise, hypertension [24]. Thus, prediction of the risk for dyslipidemia on the basis of genetic variants would be beneficial for the personalized prevention of CAD. Recent GWASs and meta-analysis on European population have identified many genes previously implicated in lipid regulation. However, validation of these loci on different global populations is still unclear.

Mulao nationality is one of the 11 minorities in Guangxi Zhuang Autonomous Region, which is a genetic feature distinctive nationality. Strict intra-ethnic marriages have been performed in this population and they have unique lifestyle as well as diet from time immemorial. Therefore, we surmised that the hereditary characteristic and some lipid-related gene polymorphisms in the Mulao population may be different from those in Han nationality.

The genotypic and allelic frequencies of *ST3GAL4* rs11220462 SNP in diverse racial/ethnic groups have not been reported. The data in the International HapMap Project’s data-base have showed that the frequencies of A allele and AA, AG genotypes were 42.0%, 15.9% and 52.3% in Han Chinese in Beijing; 30.7%, 9.1% and 43.2% in Japanese; and 15.8%, 1.7% and 0% in Yoruba; respectively. In the present study, we showed that the frequencies of A allele and AA, AG genotypes were 35.5%, 11.2% and 48.7% in Mulao; and 38.3%, 13.0% and 50.6% in Han; respectively. There were no conspicuous differences in the genotypic and allelic frequencies of the rs11220462 SNP between the Mulao and Han populations, or between males and females in the both ethnic groups. As compared with the data in the International HapMap Project’s data-base, we found that the frequencies of the A allele and AA, AG genotypes in our study populations were lower than those in Han Chinese from Beijing, which may be caused by different sample sizes and regions.

The potential association between the rs11220462 SNP and serum lipid levels in humans has been detected in a previous GWAS. Teslovich *et al*. [15] showed that the rs11220462 SNP was significant associated with LDL-C concentration (*P =* 1.20 × 10^−15^) in population of European descent. In our present study, we found that the levels of TC and LDL-C were different among the AA, AG and GG genotypes of the rs11220462 SNP in Mulao, the A allele carriers had higher serum TC, LDL-C and ApoB levels than the A allele non-carriers in the both males and females. For Han nationality, the levels of TC and TG were also different among the three genotypes in males but not in females, the subjects with AA genotype had higher TC and TG levels than the subjects with AG or GG genotype. These findings suggest that the association of rs11220462 SNP and serum lipid levels is different between the two ethnic groups, and there may be a sex-specific association of rs11220462 SNP and serum lipid parameters in the Han population. These differences may also be related to the variations in examined populations, including physical condition, environmental factors such as diet or under medication. Therefore, the association of the *ST3GAL4* rs11220462 SNP and serum lipid levels needs to be further studied with larger sample size.

There was no previous study to report the direct effect of *ST3GAL4* rs11220462 SNP on serum lipid levels. We suspected that there may be several potential mechanisms to explain the association between the rs11220462 SNP and serum lipid levels. Firstly, *ST3GAL4* encodes a member of the glycosyltransferase 29 family which was associated with increasing risk of type 2 diabetes and cardiovascular disease [19]. Secondly, Adamska *et al.*[21] reported that the *ST3GAL4* plays a key role in the synthesis of E-selectin ligands which is associated with metabolic syndrome and coronary artery calcification. Thirdly, a recent GWAS has identified several intronic SNPs including rs11220462 associated with lipid levels [15]. About 5-10% of genomic variants found in familial hypercholesterolemia are located in the introns of the candidate genes and are classified as splicing mutations [25]. More and more researcher found that intron is involved in mRNA transcription and translation regulation. Finally, microRNAs (miRNAs), a family of small non-coding RNA molecules, appear to regulate animal lipid metabolism and preadipocyte conversion to form lipid-assimilating adipocytes [26]. Recently, genetic model of beef cattle was used to analyze the association between genomic context characteristics of miRNAs (38.7% located in intron) with their function in adipose tissue [27]. The findings suggested that miRNAs play a role in the regulation of bovine adipogenesis and fat metabolism. Taken together, the rs11220462 SNP may act in an intronic variation to influence the *ST3GAL4*-associated regulatory network or specific miRNAs regulating adipogenesis. However, the biological function and detailed role of *ST3GAL4* rs11220462 SNP in lipid metabolism remain unclear and need to be further explored.

It is well realized that environmental factors such as dietary patterns, lifestyle and physical inactivity are all strongly related with serum lipid levels [24]. In the present study, multivariate linear regression analysis also showed that age, sex, BMI, alcohol consumption, cigarette smoking, and blood pressure were involved in determining serum lipid parameters in both ethnic groups. These data suggest that the environmental factors also play an important role in determining serum lipid levels in our study populations. Differences in serum lipid levels between the two ethnic groups could be related to factors such as differences in the genetic background, dietary patterns and lifestyle and their interactions. Although rice and corn are the staple food in the both ethnic groups, the people of Mulao nationality prefer cold foods along with acidic and spicy dishes. Therefore bean soy sauce and pickle vegetables become members of their most popular dishes. Meanwhile, they enjoy eating animal offals which contain abundant saturated fatty acid. For nearly 50 years it has been widely accepted that high-fat diets, particularly those contain rich saturated fatty acids, can raise the serum cholesterol concentrations as well as the risk of suffering cardiovascular disease [28]. Diet alone could be responsible for up to 2.5% of the variability on serum lipid levels [29,30]. A meta-analysis stated that diet modification is a key strategy for prevention and regression of CAD, and for every 1% decrease in energy consumed as dietary saturated fatty acid, TC decreased by 0.056 mmol/L and LDL-C by 0.05 mmol/L [31]. In addition, we also found that the percentages of smokers were higher in Mulao than in Han nationalities (*P* <0.05). Over the years, the relationship between cigarette smoking and dyslipidemia has been gotten in focus by more and more investigators. A recent study in smoking males (ages from 25 to 35 years old) has found that a significant increase in TC and LDL-C in tobacco users [32,33]. Another recent meta-analysis also reported that smoking increased TG by 0.15 mmol/L, and decreased HDL-C by 0.09 mmol/L with every 20 cigarettes smoked [34]. Therefore, the results of exposure to different lifestyle and environmental factors probably further modify the association of genetic variations and serum lipid levels in our study populations.

There are several major strengths in our study. First, the study is an investigation of a representative random sample of the Mulao population, which retains its regional and special customs in China and may be a useful subgroup for population genetic studies. Second, the sample size of the study is moderate with 700 subjects of Mulao and 694 subjects of Han Chinese. Third, a recent GWAS has reported the association between the rs11220462 SNP and serum lipid levels and our present study is the first replication of GWAS signals in the Chinese population to provide significant evidence for the association of the rs11220462 SNP with lipid traits. To interpret the findings, however, several potential limitations in our study should be acknowledged. First, all of the lifestyle, diet pattern and physical activities are the important factors for lipid regulation and prevalence of CAD, however, the cross-sectional study design limits the ability to determine the effects of them during the statistical analysis since they were self-reported and difficult to classify. Second, although we have explored the association of *ST3GAL4* rs11220462 SNP and serum lipid levels, there are still many unmeasured environmental and genetic factors such as other *ST3GAL4* SNPs and the interactions of gene-gene and/or gene-environment. Finally, the *ST3GAL4* rs11220462 SNP is an intronic variation which does not lead to a change in the amino acid sequence of the *ST3GAL4*. Thus, the effect of the rs11220462 SNP on lipid metabolism may be limited. To confirm our findings, further in-depth studies on the biological actions of *ST3GAL4* rs11220462 variation and the interactions of gene-environment are necessary.

## Conclusions

The present study showed that the genotypic and allelic frequencies of *ST3GAL4* rs11220462 SNP were not different between the Mulao and Han populations, or between males and females in the both ethnic groups. But the association of the rs11220462 SNP and serum lipid levels is different between the two ethnic groups. The A allele carriers in the both Mulao males and females had higher TC, LDL-C and ApoB levels than the A allele non-carriers. The subjects with AA genotype in Han males but not in females had higher TC and TG levels than the subjects with AG or GG genotype. These findings suggest that there may be a racial/ethnic-specific association, and/or sex-specific association between the *ST3GAL4* rs11220462 SNP and serum lipid parameters in some ethnic groups.

## Methods and materials

### Participants

Participants in the present study included 700 individuals of Mulao nationality who live in Luocheng Mulao Autonomous County, Guangxi Zhuang Autonomous Region, People’s Republic of China. There were 337 males (48.1%) and 363 females (51.9%). All of them were randomly selected from our previous stratified randomized samples [35]. The ages of the participants ranged from 15 to 93 years, with an average age of 52.56 ± 15.04 years. All of them were rural agricultural workers. In the meantime, a total of 694 Han nationality who reside in the same villages were also randomly selected from our previous stratified randomized samples. The average age of the subjects was 52.26 ± 15.02 years, which ranged from 15 to 84 years. There were 331 men (47.7%) and 363 women (52.3%). All of them were also rural agricultural workers. The whole study subjects were essentially healthy and had no evidence of any chronic illness, including hepatic, renal, or thyroid. The participants with a history of heart attack of myocardial infarction, stroke, congestive heart failure, diabetes or fasting blood glucose ≥ 7.0 mmol/L determined by glucose meter were excluded from the analyses. The participants did not take medications known to affect serum lipid levels (lipid-lowering drugs such as statins or fibrates, beta-blockers, diuretics, or hormones). Ethical approval for this study was obtained from the Ethics Committee of the First Affiliated Hospital, Guangxi Medical University. Written informed consent was provided by all participants.

### Epidemiological survey

The survey was carried out using internationally standardized methods [36]. All participants underwent a complete history, physical examination, and laboratory assessment of cardiovascular risk factors. Information on demographics, socioeconomic status, and lifestyle factors was collected with standardized questionnaires. Smoking status was categorized into groups of cigarettes per day: ≤ 20 and >20. The alcohol information included questions about the number of liang (about 50 g) of rice wine, corn wine, rum, beer, or liquor consumed during the preceding 12 months. Alcohol consumption was categorized into groups of grams of alcohol per day: ≤ 25 and > 25. Sitting blood pressure was measured three times with the use of a mercury sphygmomanometer after the subjects had a 5-minute rest, and the average of the three measurements was used for the level of blood pressure. Systolic blood pressure was determined by the first Korotkoff sound, and diastolic blood pressure was determined by the fifth Korotkoff sound. Body weight, to the nearest 50 grams, was measured by using a portable balance scale. Subjects were weighed without shoes and minimum of clothing. Height was measured, to the nearest 0.5 cm, using a portable measuring device. From these two measurements, BMI (kg/m^2^) was calculated.

### Laboratory methods

A venous blood sample of 5 mL was drawn from all individuals after an overnight (at least 12 hours) fast. The sample was divided into two parts. One part of the sample (2 mL) was collected into glass tube and allowed to clot at room temperature and used to measure serum lipid levels. Another part of the sample (3 mL) was collected into glass tube with anticoagulate solution (4.80 g/L citric acid, 14.70 g/L glucose, and 13.20 g/L tri-sodium citrate) and used to extract DNA. The levels of serum TC, TG, HDL-C, and LDL-C in the samples were determined by enzymatic methods with commercially available kits (RANDOX Laboratories Ltd., Ardmore, Diamond Road, Crumlin Co. Antrim, United Kingdom, BT29 4QY; Daiichi Pure Chemicals Co., Ltd., Tokyo, Japan). Serum ApoAI and ApoB levels were detected by the immunoturbidimetric immunoassay using a commercial kit (RANDOX Laboratories Ltd.). All determinations were performed with an autoanalyzer (Type 7170A; Hitachi Ltd., Tokyo, Japan) in the Clinical Science Experiment Center of the First Affiliated Hospital, Guangxi Medical University [24].

### DNA preparation and genotyping

Genomic DNA was isolated from peripheral blood leukocytes using the phenol-chloroform method [35]. The extracted DNA was stored at −20°C until analysis. Genotyping of the rs11220462 SNP was performed by polymerase chain reaction and restriction fragment length polymorphism (PCR-RFLP). PCR amplification was performed using 5′-CGATGCTATCCGATGAAC-3′ and 5′-TCACTGTAACCTCCACCTC-3′ (Sangon, Shanghai, People’s Republic of China) as the forward and reverse primer pairs; respectively. Each amplification reaction was performed in a total volume of 25 mL reaction mixture, including 100 ng (2 μL) of genomic DNA, 0.75 μL of each primer (10 μmol/L), 12.5 μL 2 × *Taq* PCR MasterMix (constituent: 0.1 U *Taq* polymerase/μL, 500 μM dNTP each, 20 mM Tris–HCl, pH 8.3, 100 mM KCL, 3 mM MgCl_2_, and stabilizers), and 9.0 μL nuclease-free water. After initial denaturizing at 94°C for 5 min, the reaction mixture was subjected to 32 cycles of denaturation at 94°C at 45 s, annealing at 57°C for 35 s and extension 35 s at 72°C, followed by a final 8 min extension at 72°C. After electrophoresis on a 2% agarose gel with 0.5 μg/mL ethidium-bromide, the amplification products were visualized under ultraviolet light. Then the *Sat*I restriction enzyme of 2.0 U was added directly to the reaction system consisting of PCR products (5 μL), 9 μL of nuclease-free water and 1 μL of 10 × buffer solution and digested at 37°C for 8 hours. After restriction enzyme digestion of the amplified DNA, genotypes were identified by electrophoresis on 2.0% agarose gel and visualized with ethidium-bromide staining ultraviolet illumination. The PCR produced a 437-bp fragment including one *Sat*I recognition site for the G allele (Additional file 1: Figure S1). The G allele can be cleaved whereas the A allele cannot be digested (Additional file 2: Figure S2). Therefore, the GG genotype is homozygote for the presence of the site (band at 340- and 97-bp), AG genotype is heterozygote for the absence and resence of the site (bands at 437-, 340- and 97-bp), and AA genotype is homozygote for the absence of the site (bands at 437-bp). Genotypes were scored by an experienced reader blinded to epidemiological data and serum lipid levels. The genotypes detected by PCR-RFLP were also confirmed randomly by direct sequencing (Additional file 3: Figure S3, Sangon Biotech Co., Ltd., Shanghai, People’s Republic of China).

### Diagnostic criteria

The normal values of serum TC, TG, HDL-C, LDL-C, ApoAI, ApoB levels and the ratio of ApoAI to ApoB in our Clinical Science Experiment Center were 3.10-5.17, 0.56-1.70, 1.16-1.42, 2.70-3.10 mmol/L, 1.20-1.60, 0.80-1.05 g/L, and 1.00-2.50; respectively [37]. The individuals with TC > 5.17 mmol/L, and/or TG > 1.70 mmol/L were defined as hyperlipidemic [35]. Hypertension was diagnosed according to the criteria of 1999 World Health Organization-International Society of Hypertension Guidelines for the management of hypertension. Hypertension was defined as an average systolic blood pressure (SBP) ≥ 140 mmHg, and/or an average diastolic blood pressure (DBP) ≥ 90 mmHg, and/or self-reported current treatment for hypertension with antihypertensive medication [37]. The diagnostic criteria of overweight and obesity were according to the Cooperative Meta-analysis Group of China Obesity Task Force. Normal weight, overweight and obesity were defined as a BMI < 24, 24–28, > 28 kg/m^2^; respectively [38].

### Statistical analysis

The statistical analyses were done with the statistical software package SPSS16.0 (SPSS Inc., Chicago, Illinois). Quantitative variables were expressed as mean ± standard deviation (serum TG levels were presented as medians and interquartile ranges). Qualitative variables were expressed as percentages.The difference in general characteristics between two ethnic groups was tested by the Student’s unpaired *t*-test. Allele frequency was determined via direct counting, and the standard goodness-of-fit test was used to test the Handy-Weinberg equilibrium. Difference in genotype distribution between the groups was obtained using the chi-square test. The association of genotypes and serum lipid parameters was tested by analysis of covariance (ANOVA). Age, sex, BMI, blood pressure, alcohol consumption, cigarette smoking and blood glucose were included in the statistical models as covariates. Multiple linear regression analyses adjusted for age, sex, BMI, blood pressure, alcohol consumption, cigarette smoking and blood glucose were also performed to assess the association of serum lipid levels with genotypes (AA = 0, AG = 1, GG = 2) and several environment factors. A *P* value of less than 0.05 was considered statistically significant.

## Competing interests

The authors declare that they have no competing interests.

## Authors’ contributions

QZL participated in the design, undertook genotyping, performed the statistical analyses, and drafted the manuscript. RXY conceived the study, participated in the design, carried out the epidemiological survey, collected the samples, and helped to draft the manuscript. TG, JW, JQS, SWS and GYS collaborated to the genotyping. JZW, CWL and SLP carried out the epidemiological survey and collected the samples. All authors read and approved the final manuscript.

## Supplementary Material

Additional file 1: Figure S1 Electrophoresis of PCR products of the samples. Lane M, 100 bp marker ladder; lanes 1–5, samples. The 437 bp bands are the target genes. Click here for file

Additional file 2: Figure S2 Genotyping of the rs11220462 SNP. Lane M, 100 bp marker ladder; lane 1 and 2, AA genotype (437 bp); lanes 3 and 4, AG genotype (437- ,340- and 97-bp); and lanes 5 and 6, GG genotype (340-bp). The 97 bp fragment was invisible in the gel owing to its fast migration speed. Click here for file

Additional file 3: Figure S3 A part of the nucleotide sequence of the 11220462 SNP A > G polymorphism. (A) AA genotype, (B) AG genotype and (C) GG genotype. Click here for file
