# Supplementary figures and images for: Association of the ST3GAL4 rs11220462 polymorphism and serum lipid levels in the Mulao and Han populations
Source: Lipids Health Dis. 2014 Aug 3;13:123. doi: 10.1186/1476-511X-13-123 (PMC4237880; doi:10.1186/1476-511X-13-123)

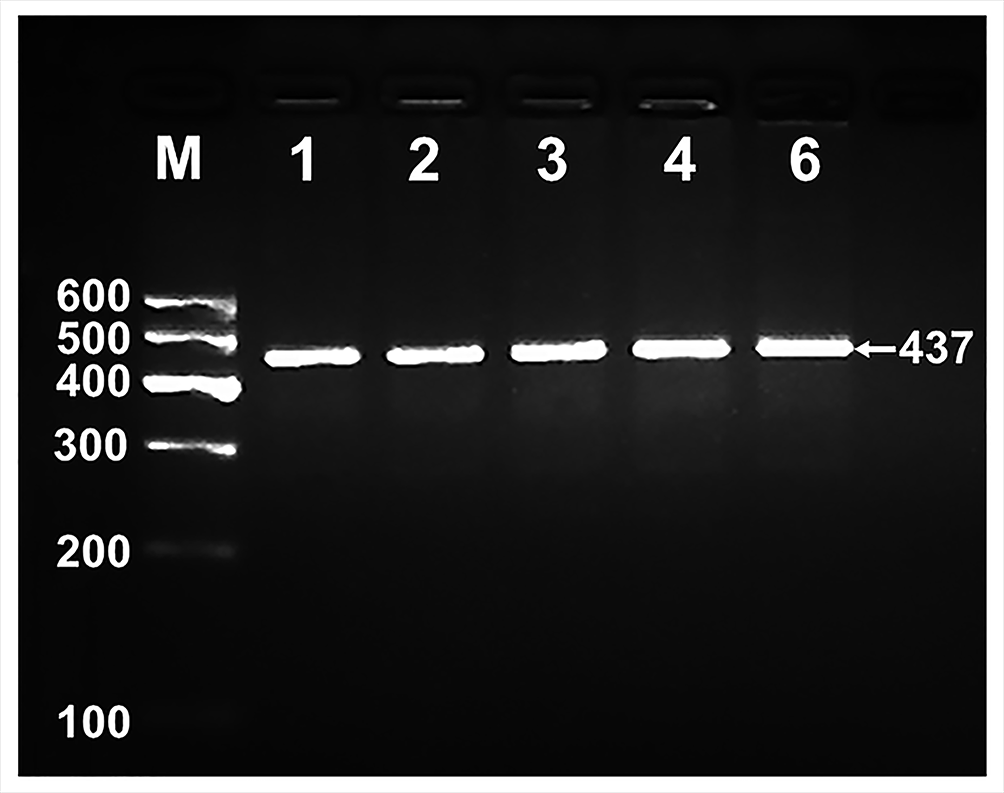

Supplement: Additional file 1: Figure S1 — Electrophoresis of PCR products of the samples. Lane M, 100 bp marker ladder; lanes 1–5, samples. The 437 bp bands are the target genes. [file 1476-511X-13-123-S1.tiff]

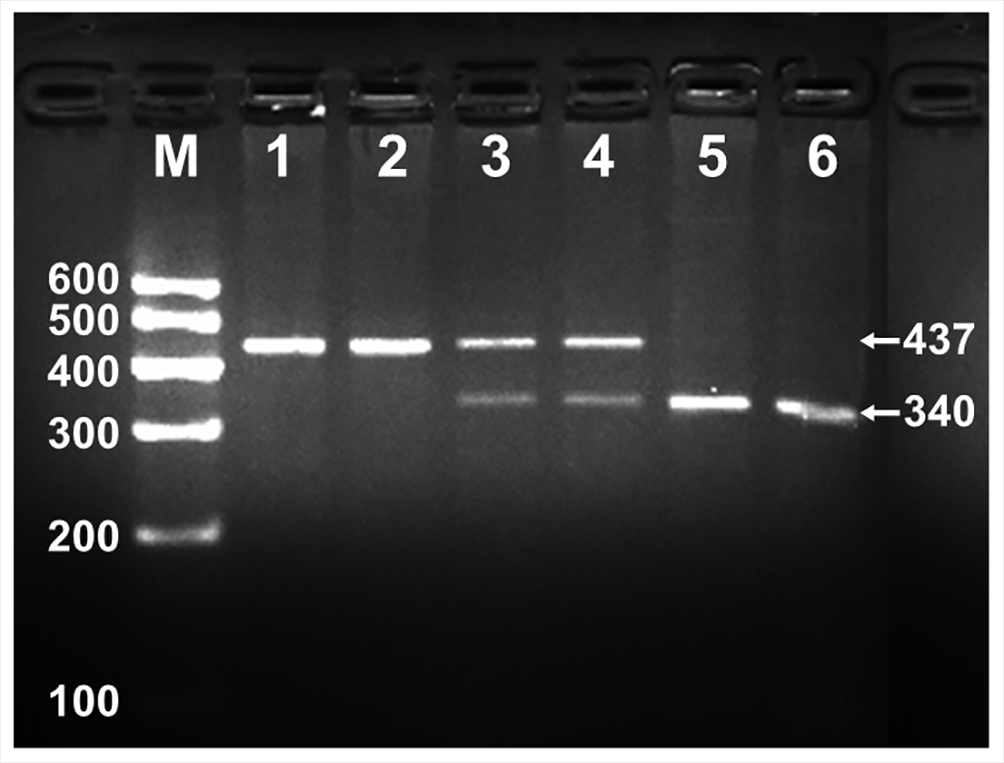

Supplement: Additional file 2: Figure S2 — Genotyping of the rs11220462 SNP. Lane M, 100 bp marker ladder; lane 1 and 2, AA genotype (437 bp); lanes 3 and 4, AG genotype (437- ,340- and 97-bp); and lanes 5 and 6, GG genotype (340-bp). The 97 bp fragment was invisible in the gel owing to its fast migration speed. [file 1476-511X-13-123-S2.tiff]

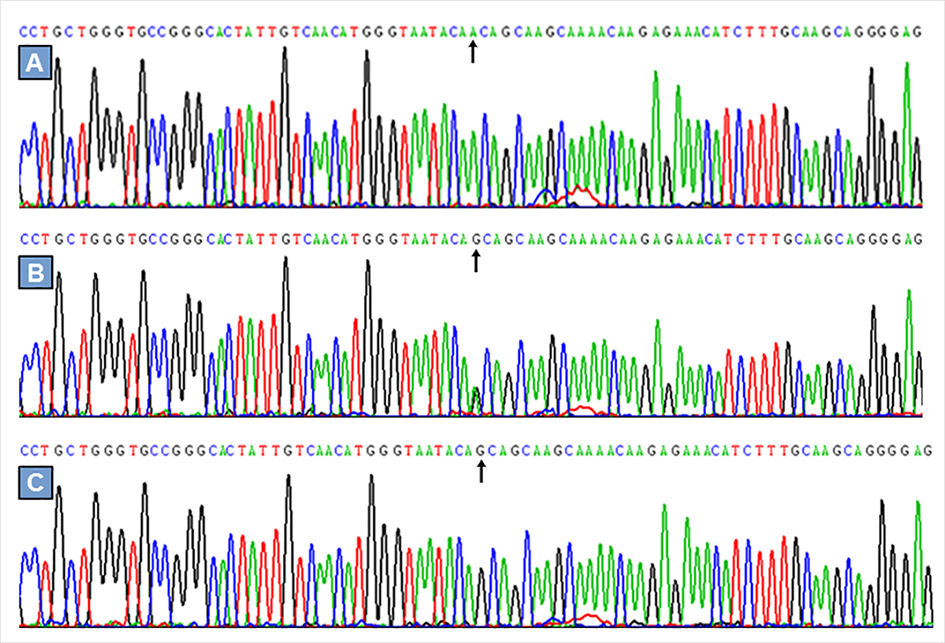

Supplement: Additional file 3: Figure S3 — A part of the nucleotide sequence of the 11220462 SNP A > G polymorphism. (A) AA genotype, (B) AG genotype and (C) GG genotype. [file 1476-511X-13-123-S3.tiff]
